# Dutch Forensic Flexible Assertive Community Treatment: Operating on the Interface Between General Mental Health Care and Forensic Psychiatric Care

**DOI:** 10.3389/fpsyg.2021.708722

**Published:** 2021-09-22

**Authors:** Marjam V. Smeekens, Fedde Sappelli, Meike G. de Vries, Berend H. Bulten

**Affiliations:** ^1^Department Assessment, Research and Professional Development, Pompefoundation, Nijmegen, Netherlands; ^2^Faculty of Social Sciences, Radboud University, Nijmegen, Netherlands

**Keywords:** forensic assertive community treatment, disruptive behavior, patient characteristics, mental illness, offending, responsivity

## Abstract

In the Netherlands, *Forensic Flexible Assertive Community Treatment* (ForFACT) is used as a specialized form of outpatient intensive treatment. This outreaching type of treatment is aimed at patients with severe and long lasting psychiatric problems that are at risk of engaging in criminal behavior. In addition, these patients often suffer from addiction and experience problems in different areas of their life (e.g., financial debt, unemployment, or lack of daytime activities). The aim of this exploratory study was to gain more insight into the characteristics of the ForFACT patient population. More knowledge about these patients may enhance the effectiveness of ForFACT and therefore (further) reduce the risk of recidivism. Data on 132 ForFACT patients were gathered by studying electronic patient records, criminal records, and by conducting semi-structured interviews with practitioners and patients. Additionally, as part of a cognitive screening, two screening instruments were conducted to gain insight into intelligence and possible mild cognitive impairments. This article gives a broad description of the ForFACT patient population, including demographic data and context variables, diagnostics, recidivism risk and offense history, and aspects related to care. Furthermore, several recommendations are given to further improve ForFACT. Based on the results it can be concluded that the ForFACT patient population shows a high degree of diversity in complex care needs and responsivity issues. Therefore, this article highlights the necessity for ForFACT to collaborate with other mental health institutions, as well as probation officers, and forensic or criminal justice institutions. Moreover, it is important to continually check the inclusion and exclusion criteria when admitting patients to ForFACT, and to examine whether ForFACT is still the most adequate care for patients or if they need to be referred. In addition, the results emphasize the importance of cognitive screening for forensic outpatients. Finally, this study zooms in on the interface between forensic psychiatric care and general mental health care.

## Introduction

In the last 60 years, the focus of psychiatric care in Western countries transitioned from conventional institutional settings to community-based services (Novella, 2008). This process of deinstitutionalization led to the closure of many psychiatric hospitals, causing patients with severe mental health problems to be discharged. In response to the needs of these patients to receive treatment and support within the community, Assertive Community Treatment (ACT) was developed in the early 1970s (Marshall and Lockwood, 1998). ACT teams are multidisciplinary, have a low and shared caseload, are available 24 h a day, and operate on an outreaching level, which means that the practitioners visit the patient in their own home or living environment for treatment (Marshall and Lockwood, 1998; Bond et al., 2001). ACT has been proven effective in reducing hospitalization, improving community tenure and is established as an evidence-based practice nowadays (Aagaard et al., 2017; Thorning and Dixon, 2020).

In the Netherlands, an adaptation of the ACT model was developed, called *flexible* ACT (Van Veldhuizen, 2007). This Dutch treatment model focuses on supporting and treating a broader group of patients with a prolonged need of mental health care. Flexible ACT teams are able to flexibly switch between two types of care, depending on the changeable needs, circumstances, and conditions of a patient (Nugter et al., 2016). The teams provide assertive outreach (ACT principles) for the most severely ill psychiatric patients and individual case management for patients who are relatively stable (Van Veldhuizen, 2007). Therefore, flexible ACT teams ensure the continuity of care through their ability to (temporarily) (de)intensify treatment.

The deinstitutionalization within psychiatric care in Western countries partly led to a process of transinstitutionalization, meaning that individuals who previously resided in psychiatric hospitals, were frequently found in prisons over time (Prins, 2011; Schildbach and Schildbach, 2018). According to the (also criticized) Penrose hypothesis, the number of psychiatric hospital beds is inversely related to the size of prison populations (Penrose, 1939). Many countries reported an overrepresentation of severely mentally ill individuals in prisons and the criminal justice system over the past few years (Lamberti and Weisman, 2010; Prins, 2014; Juriloo et al., 2017; Favril and Dirkzwager, 2019). Several studies state that this increase in criminalization is indeed associated with the decrease of hospital beds, supporting the Penrose hypothesis (Mundt et al., 2015; Toynbee, 2015; Schildbach and Schildbach, 2018). However, other studies found no evidence for such a direct relationship (Large and Nielssen, 2009; Bluml et al., 2015).

In order to prevent incarceration, justice-involved individuals with mental health problems are in need of preventive care within the community. In addition, if these individuals do get sentenced to prison, they should be provided with an appropriate type of aftercare. However, successful (re)integration into the community for justice-involved individuals with mental illness seems to be difficult (Cuddeback et al., 2020). For example, they experience difficulties in accessing supportive housing, finding employment, and accessing the appropriate type of (follow-up) treatment (Mallik-Kane and Visher, 2008). Furthermore, there has been an increase in reported incidents by the police of people with mental illness (Livingston, 2016). These studies and reports show that there is a group of patients with complex psychopathology and forensic problems that are in need of (preventive) community-based specialized care.

Considering ACT has proven to be an effective community-based treatment for psychiatric patients, the question arose whether this treatment model would also be effective in treating forensic psychiatric patients. However, research showed that ACT is not effective in reducing forensic outcomes, such as arrests and incarcerations (Calsyn et al., 2005; Cosden et al., 2005). Therefore, an adaption of the ACT-model was developed in the beginning of the twenty-first century, called *Forensic Assertive Community Treatment* (FACT) (Lamberti and Weisman, 2010). In contrast to regular ACT teams, which primarily focus on increasing well-being of patients, FACT teams also focus on reducing and preventing recidivism, and improving safety in society (Cuddeback et al., 2020). To achieve this, FACT teams focus on risk management and relapse prevention by applying various forensic tools, such as risk assessment and offense analysis, upon which treatment goals are based. Furthermore, FACT collaborates with probation officers, forensic clinics, the police, the public prosecutor's office, and prisons in order to provide specialized forensic care.

Since the primary goal of FACT is to prevent criminal behavior, the FACT method corresponds to the *Risk-Need-Responsivity* (RNR) model (Lamberti and Weisman, 2010). According to the RNR model, a forensic psychiatric intervention will be effective if it meets three principles (Andrews et al., 1990). The *risk* principle states that the intensity of care should match the risk of a patient to reoffend; patients with a high risk of reoffending will need a higher intensity of care. The *need* principle emphasizes the importance of assessing criminogenic needs (factors that influence (re)offending) when defining and implementing treatment. The *responsivity* principle says that the type of treatment should be tailored to the learning style, motivation, and capacities of a patient.

Several studies looked into the question whether FACT is an effective type of treatment for forensic psychiatric patients. In contrast to ACT, FACT has indeed proven to be effective in preventing arrest and incarceration (Lamberti et al., 2004; Marquant et al., 2018). Two randomized controlled trials compared FACT vs. treatment as usual and found significant improvements on several judicial outcome measurements due to FACT (such as fewer convictions for new crimes and less time in jail) (Cusack et al., 2010; Lamberti et al., 2017). These studies also found that FACT patients made greater use of outpatient mental health services than patients who received treatment as usual.

In the Netherlands, flexible ACT was combined with FACT, resulting in *Forensic Flexible Assertive Community Treatment* (ForFACT) (Place et al., 2011). Like flexible ACT, ForFACT combines individual case management and ACT principles in response to the changeable care needs of patients (though aimed at forensic patients). The ForFACT patient population is often characterized by a long history of (forensic) care, multiple clinical admissions, and relapses in forensic psychiatric problems (Place et al., 2011). ForFACT is meant for patients that, because of their complex problems, do not benefit from care offered by a forensic outpatient clinic. ForFACT patients are sensitive to crises and therefore profit from an outreaching type of care, where treatment can be intensified depending on the current needs of a patient.

Over the past few years, there has been a rapid increase in the number of ForFACT teams in the Netherlands (Kroon et al., 2016). However, little is known about the effectiveness of ForFACT. One longitudinal study has been conducted, including eight Dutch ForFACT teams (Neijmeijer et al., 2017). After 1 year of treatment, ForFACT patients significantly improved on psychological and social functioning, were detained less often, and were considered to be less likely to reoffend. Although, it has to be noted that no control group was included to compare these results. Nevertheless, the results on the effectiveness of ForFACT sound promising.

In order to enhance the effectiveness of ForFACT and thereby following the three RNR-principles, there should be enough knowledge about what characterizes this patient population. Research shows that there is a weak association between the indication criteria for ForFACT and risk factors included in the Short-Term Assessment of Risk and Treatability (START) (Kusters et al., 2018). For ForFACT patients, compared to other delinquents, these clinical indication criteria seem to have a more central role at predicting reoffending than criminogenic factors. Previous studies described several ForFACT patient characteristics (Neijmeijer et al., 2017; Kusters et al., 2018). However, more knowledge about the (criminogenic) needs and the responsivity of ForFACT patients is needed and may help to further implement tailored care for these individuals.

In theory, ForFACT offers treatment to forensic patients, but in practice, the distinction between forensic patients and regular psychiatric patients is often not clear due to the large variance in patient characteristics and situational factors. This makes it difficult to determine which type of treatment (forensic or regular mental health care) should be indicated, and as a consequence some patients can get “stuck” between the two systems (The Netherlands Institute for Forensic Psychiatry Psychology, 2016). For example, it seems there is a group of ForFACT patients who do not display actual criminal behavior, but they do show some kind of disruptive behavior (e.g., incidents toward staff, failing to keep agreements, or nuisance). The question arises whether these patients belong to ForFACT, or whether they should receive treatment from a regular flexible ACT team. In addition, does the type of treatment ForFACT offers, correspond to the needs of these different types of patients?

In an effort to answer these questions, the main goal of this exploratory study was to gain insight into the characteristics of the complex ForFACT patient population, their (criminogenic) needs, and issues concerning responsivity. Offering an appropriate type of care to forensic patients, in line with the RNR model, is of great importance to decrease the risk of criminal behavior and individual suffering, as well as to secure societal safety. Information was gathered about patients who received treatment within a Dutch ForFACT facility. Based on the results, several recommendations are given to further improve ForFACT.

## Materials and Methods

### ForFACT

This research study took place at a Dutch outpatient mental health care facility: Kairos (part of the Pompefoundation). Kairos offers treatment to forensic psychiatric patients and consists of three outpatient clinics and two ForFACT teams (in the region Arnhem and Tiel in the Netherlands). Both teams have been screened for model fidelity by the CCAF, the Dutch Certification Centre for ACT and FACT teams, by using the ForFACT scale (Bähler et al., 2019). By means of this scale, Dutch ForFACT teams are, among other things, screened for provider/recipient ratio, staff composition, expertise on somatic problems/addiction/intellectual disability, and level of outreach. The ForFACT teams of Kairos received a certification from the CCAF.

ForFACT offers treatment to adults that suffer from a psychological disorder and have committed one or more (serious) offenses for which they received criminal charges. In the Netherlands, there is a policy stating that ForFACT may also treat patients who are expected to show an elevated risk of criminal behavior if no forensic treatment would be offered, for instance for patients with serious socially disruptive behavior. Thus, the Dutch ForFACT teams partly operate in a preventive manner, since patients can also be referred to ForFACT without a current conviction (e.g., by their general practitioner). A combination of social exclusion, expulsion from regular treatment, complex psychopathology, limited social network or a strong criminal network often lead to these people relapsing within criminality, or committing a crime for the first time. This subgroup of patients is treated without a decision of the court. Moreover, ForFACT patients often suffer from addiction, mild cognitive impairments and experience problems in different areas of their life (e.g., financial debt, unemployment, or lack of daytime activities).

People are excluded from treatment at Kairos ForFACT when they are younger than 18 years old, do not have a correspondence address, or do not have health insurance (if a correspondence address and health insurance can be arranged within a short period of time, there are possibilities to start with treatment, for instance for homeless patients). Furthermore, if patients refuse to give permission to ForFACT to request treatment information from other/past mental health care facilities or judicial reports, they will also be excluded. In case of extreme addiction or psychotic problems, a patient can be treated in collaboration (e.g., sharing expertise or actually offering two types of treatment) with other mental health care institutions (such as addiction treatment, a crisis team or a regular flexible ACT team). Lastly, ForFACT coordinates treatment together with other forensic services, mental health services and municipalities within a communication platform, the so called Dutch “*veiligheidshuizen*.” Depending on the needs of the patient, this communication platform also includes, for instance, probation officers, community police officers, and the Dutch Custodial Institutions Agency.

### Participants

Based on their own caseload, the ForFACT practitioners determined which patients were eligible to participate in the study. Patients were excluded from participation if they were in detention or in (psychological) crisis. In total, 223 ForFACT patients were approached for participation in this research study. Eventually, 132 patients agreed to participate, of which 18 patients limited their permission to only study his/her electronic patient record and criminal record. Demographic information about the study sample can be found in the result section.

### Instruments

#### Electronic Patient Records

An extensive study of the electronic patient records of the 132 participating patients was conducted. More specifically, information was gathered from the intake interview report, treatment plan, diagnostics, and medication files. Information about patient characteristics that was considered relevant for this study, but could not always be retrieved from the electronic patient records, was acquired by interviewing the ForFACT practitioners and patients. Based on information that was found in the electronic patient records, a timeline was created in Microsoft Word for each patient that participated in the interviews. Since ForFACT patients are often characterized by a long history of (forensic) care, this timeline represented the history of care (type of care, starting date, and duration). The timeline contained previous treatments before ForFACT, over a time span of 10 years. This time span was chosen because of the availability of information in the electronic patient records, and because 10 years was considered to be a reasonable time frame for patients to be able to recall events from their memory during the interview.

#### Forensic Outpatient Risk Evaluation

By studying the electronic patient records, data were gathered concerning recidivism risk at the moment of the intake procedure from 111 ForFACT patients. The Dutch FORE (Forensic Outpatient Risk Evaluation) is used by ForFACT as a risk assessment tool/instrument during the intake procedure and subsequently every 6 months (Van Horn et al., 2016). The main goal of the FORE is to give an estimation of the risk to reoffend and to monitor changes in risk factors during treatment. The FORE consists of 17 items and measures two types of risk factors: 6 static risk factors (unchangeable characteristics, e.g., age of first contact with the police) and 11 dynamic risk factors (changeable through intervention). Each item has a scoring range from 0 to 4 (5-point scale), were zero represents a potential protection (absence of risk) and four represents serious transgressive behavior/attitude or situation (high risk). After administering the FORE, a clinical estimation of the recidivism risk of a patient is made: very low, low, moderate, high, or very high. Subsequently, the individual dynamic risk factor are addressed and incorporated into the patient's treatment plan and treatment goals. The psychometric properties of the FORE have been studied and seem to be sufficient (Eisenberg et al., 2020).

#### Interviews With ForFACT Patients

To gain more insight into the history of care of patients, the timeline with history of care (based on the electronic patient record) was shown to the patients on printed-paper. Patients were asked about their history of care over the past 10 years: the type of care, the date, duration and the overall satisfaction with the received treatment (positive, negative or neutral). The answers the patients gave to the interview questions were added to the printed timeline.

#### Cognitive Screening

To gain insight into the cognitive vulnerabilities of the ForFACT sample, two screening instruments were conducted. One instrument was the Dutch version (8.1) of the Montreal Cognitive Assessment (MoCA), which is a validated screening instrument for mild cognitive impairments (MCI) (Thissen et al., 2010). This instrument assesses multiple cognitive domains; these are executive functioning, visuospatial abilities, attention, language, abstract reasoning, memory, and orientation. The MoCA consists of 10 items and has a scoring range from 0 to 30. A sum score of 25 or lower might indicate the presence of MCI. The second instrument was the Screener for Intelligence and Learning Disability (SCIL), which is a validated Dutch screening instrument for intellectual disabilities (Kaal et al., 2015). The SCIL consists of 14 items and has a scoring range from 0 to 28. A sum score of 20 or lower might indicate the presence of an intellectual disability. The use of this cut-off score is recommended when testing mentally ill detainees (Van Esch et al., 2020). Both the MoCA and the SCIL take ~10 min to administer and are explicitly meant for screening. To determine whether a patient has actual MCI and/or an intellectual disability, further diagnostic research is necessary.

#### Criminal Records

In the Netherlands it is possible, under certain conditions and by following the privacy laws, to request official criminal records at the judicial information service (*Justitiële Informatiedienst—JustId*, part of the Dutch Ministry of Justice and Security). For purposes of this research, official criminal records of the participating ForFACT patients were requested. These criminal records were used to make a timeline for each patient with their offense history, from the moment study was conducted until 10 years back in time. These timelines contained information about the date, type, and number of criminal offenses.

### Procedure

The Internal Scientific Committee of the Pompefoundation approved this study protocol. Data collection took place from January 2019 until August 2019. The ForFACT practitioners approached the eligible patients to participate in the study. These patients received oral and written information about the study, and an informed consent form from their practitioner. When a ForFACT patient gave informed consent to participate, his/her electronic patient record and criminal record were studied. Furthermore, if a patient also gave informed consent to participate in the interview, the timeline containing history of care was created. Consequently, an appointment with the patient and his/her practitioner was made to conduct the interview and administer the cognitive screening. Before the interview with the patient was conducted, the ForFACT practitioner was interviewed regarding the electronic patient records. Most of the interviews with the patients were conducted in the patient's own home or living environment, in presence of his/her practitioner. Some patients received treatment at the outpatient clinic and were therefore interviewed at the outpatient clinic. For a few patients the interview was conducted in multiple appointments, for example, due to a patient's limited attention span.

At the beginning of the interview, the patient was reminded of the goal, procedure, and duration of the interview. First, the patient was asked about the missing variables that could not always be retrieved from the electronic patient records. Secondly, the patient answered questions about his/her history of care. The answers the patient gave, were written on the printed timeline by the researcher. After finishing the interview, the cognitive screening was administered. If the patient completed the interview and the cognitive screening, he/she was thanked for participation and was given 15 euros as a reimbursement.

### Data Preparation and Data Analysis

The information that was gathered from the electronic patient records, criminal records, and the interviews was scored. During intensive meetings among the researchers and before any data-analyses were conducted, the variables were categorized. The information gathered from the electronic patient records, criminal records and interviews was used as a starting point for categorization (to make sure categories were in line with the available data in the electronic patient records). For “psychiatric diagnosis,” the Diagnostic and Statistical Manual of Mental Disorders (DSM, 5th edition) classification system was used (which is also used by ForFACT) (American Psychiatric Association, 2013). The inter-rater reliability was calculated to measure the degree of agreement between three researchers concerning the categorization of the variables “care need defined by the patient” and “treatment goal defined by the practitioner.” There was an almost perfect agreement concerning the categorization of care needs (κ = 0.80, *p* ≤ 0.001) and a substantial agreement concerning the categorization of treatment goals (κ = 0.55, *p* ≤ 0.001).

Patient data were pseudonymized, meaning that names were replaced with research codes. Data preparation and data-analysis were performed using IMB SPSS (25th version). Due to the exploratory nature of this research study, the data analysis mostly consisted of data exploration through descriptive statistics. However, to gain more insight into the associations and relationships between variables, additional analyses were conducted (of which the statistical assumptions were checked). The association between recidivism risk and the number of convictions was measured by calculating the Spearman's correlation coefficient. To find out about the difference between the mean scores of patients on dynamic risk factors and static risk factors, a Paired Sample *t*-test was conducted. Moreover, a non-parametric test (Mann-Whitney *U*-test) was conducted to test the difference in the number of convictions between patients with a very low to moderate risk of reoffending and a high to very high risk of reoffending. Furthermore, the Spearman correlation coefficient was calculated to measure the relationship between the current number of diagnoses and the number of treatments received in the past, and to measure the association between the sum scores on the MoCA and the sum scores on the SCIL. Some variables contained missing data, for these variables the total sample size was <132. These variables were financial debt (*n* = 128), financial guardianship (*n* = 28), medication (*n* = 131), MoCA (*n* = 112), SCIL (*n* = 108), and FORE (*n* = 111). The deviations regarding the total sample size for these variables are also mentioned in the result section.

## Results

### Demographics and Context

The ForFACT patients included in the study were 18 to 72 years old (*M* = 40.3, *SD* = 11.3) and the majority of patients was male (88%). Approximately one-fourth of the patients (26%) had a non-Dutch cultural background, meaning that the patient or his/her parents were not born in the Netherlands. When looking at employment rates, 61% of the patients was unemployed, 24% had a paid job, 8% of the patients was unfit for work, and 7% did voluntary work, had sheltered employment, or was studying. Furthermore, 32% of the patients had no further education after primary school. Regarding the financial situation of the participants: 63% of the patients had financial debt (*n* = 128) and 53% of the patients had a type of financial guardianship (*n* = 128) (e.g., full guardianship, or limited guardianship). Finally, 35% of the patients were in a romantic relationship and almost half of the patients had one or more children (46%).

### Diagnostics

**Figure 1:**
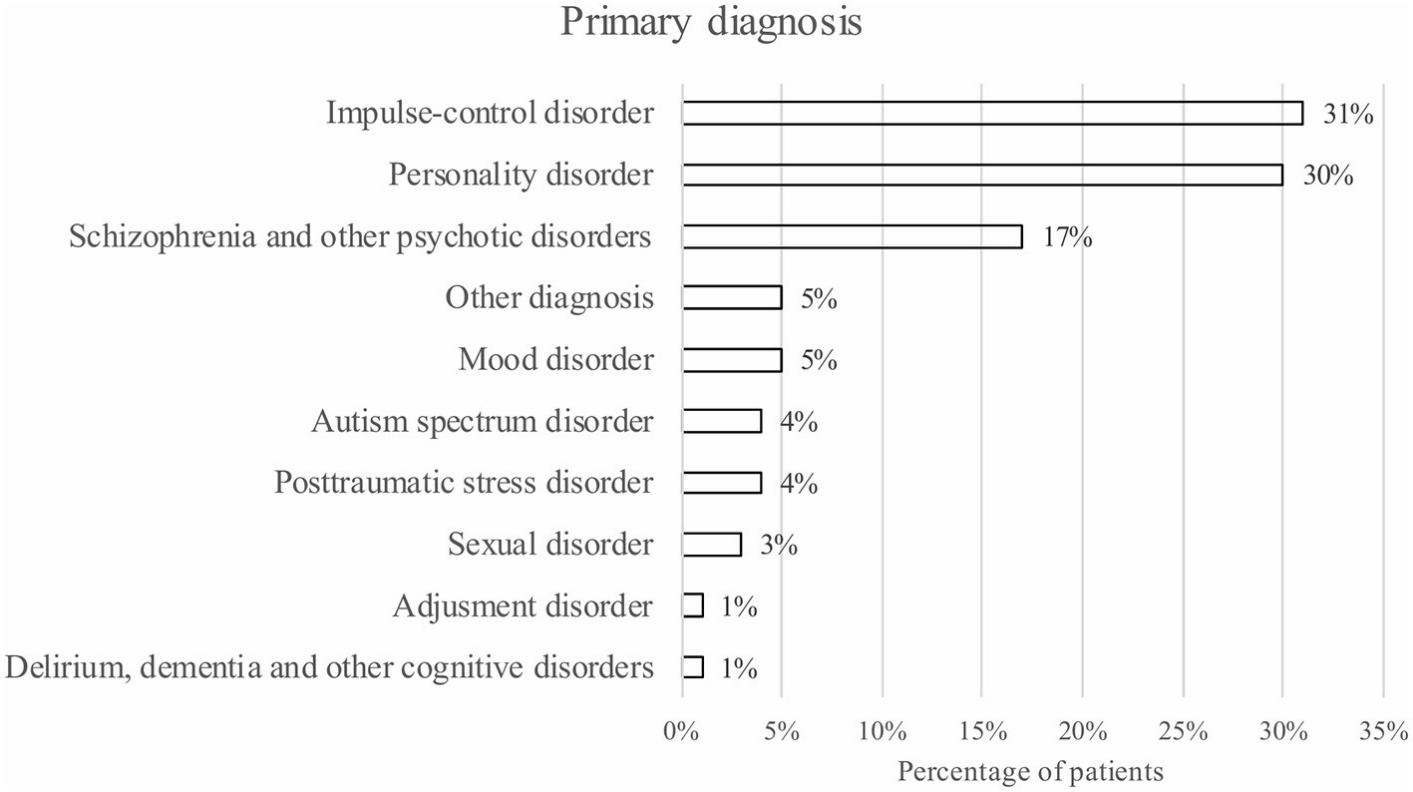
The primary diagnoses of the ForFACT patients (*n* = 132).

The most frequent primary diagnoses were impulse-control disorder (31%), personality disorder (30%), and schizophrenia and other psychotic disorders (17%) (see Figure 1). Besides a primary diagnosis, patients had (multiple) additional diagnoses. The most frequent additional diagnoses were V codes, i.e., other conditions that may be a focus of clinical attention (77%), substance-related disorders (61%), personality disorders (47%), and intellectual disabilities (17%). On average, the participating ForFACT patients had five different diagnoses (including primary diagnosis, additional diagnoses, and V codes) (*M* = 5.3, *SD* = 1.9).

The ForFACT practitioners indicated that 70% of the patients experienced problems related to substance abuse during ForFACT, of which 61% met the criteria for a DSM substance-related disorder. With regard to the type of substances, 38% of the patients experienced problems related to the use of cannabis, 33% to alcohol, and 15% to cocaine. In addition, 83% of the patients experienced addiction problems in the past 10 years (before ForFACT).

Use of medication was found in 76% of the patients (*n* = 131), of which 16% did not show medication adherence. Most patients received antipsychotics (46%) followed by sleeping pills or sedatives (39%) (*n* = 131). Nearly one out of 10 patients (9%) had a high risk of suicidality. Besides psychological problems, 57% of the participating patients had medical/somatic problems, of which 27% suffered from a chronic disability (an organ dysfunction such as asthma, cardiovascular disease or diabetes), 25% had “other” disabilities (e.g., fatigue, migraine, or dental problems), and 12% had a movement disability (e.g., arthrosis, rheumatism, or tremors).

### Cognitive Functioning

With regard to the cognitive screening, 63% of the participating ForFACT patients had a sum score of 25 or lower on the MoCA (*n* = 112). Which means that these patients had an indication for the presence of mild cognitive impairments (MCI). Furthermore, 67% of the patients had a sum score of 20 or lower on the SCIL (*n* = 108). These patients presumably had an intellectual disability. There was a strong positive correlation between the sum scores of patients on the MoCA and the SCIL [*r*_*s*__(108)_ = 0.66, *p* ≤ 0.001]. More than half of the patients (54%) had a sum score below the cut-off score on the MoCA as well as on the SCIL. Another indication for MCI or intellectual disability might be language proficiency. The ForFACT practitioners indicated that almost one-third (31%) of the patients had an insufficient to moderate Dutch language proficiency. Meaning that they, in general, did not have sufficient Dutch reading, writing, and listening skills.

### Recidivism Risk and Offense History

**Figure 2:**
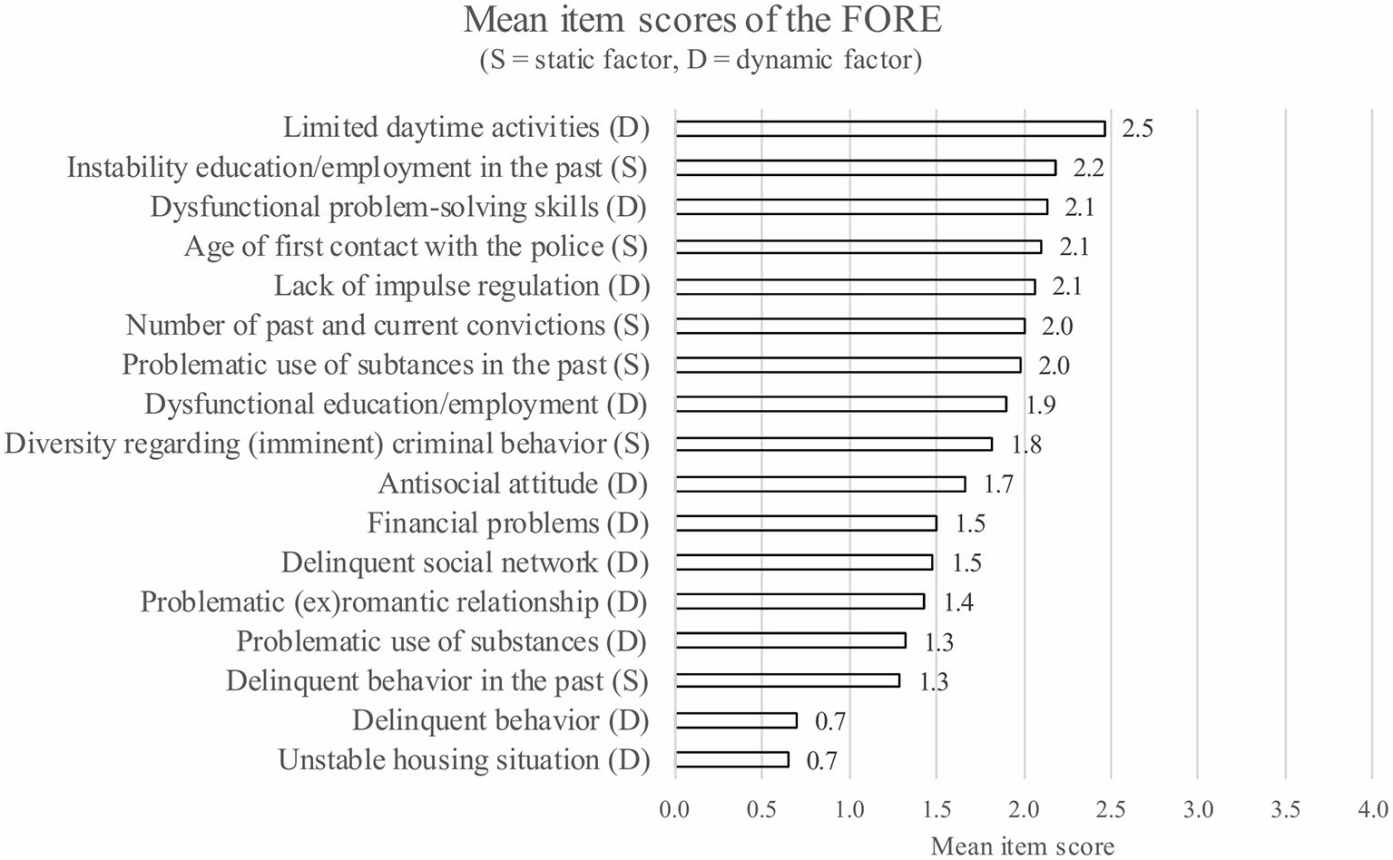
The average item scores of the ForFACT patients on the FORE (*n* = 111).

The clinical estimation of the recidivism risk of the participating ForFACT patients (during the intake procedure), based on the FORE, was as follows: 6% had a very low risk, 26% had a low risk, 26% had a moderate risk, 34% had a high risk, and 7% had a very high risk to reoffend (*n* = 111). In addition, on average, ForFACT patients scored relatively low on dynamic risk factors (*M* = 1.6, *SD* = 0.7) compared to static risk factors (*M* = 1.9, *SD* = 0.9), [*t*_(105)_ = 3.94, *p* ≤ 0.001]. Figure 2 shows the mean items scores of the participating patients on the FORE.

**Figure 3:**
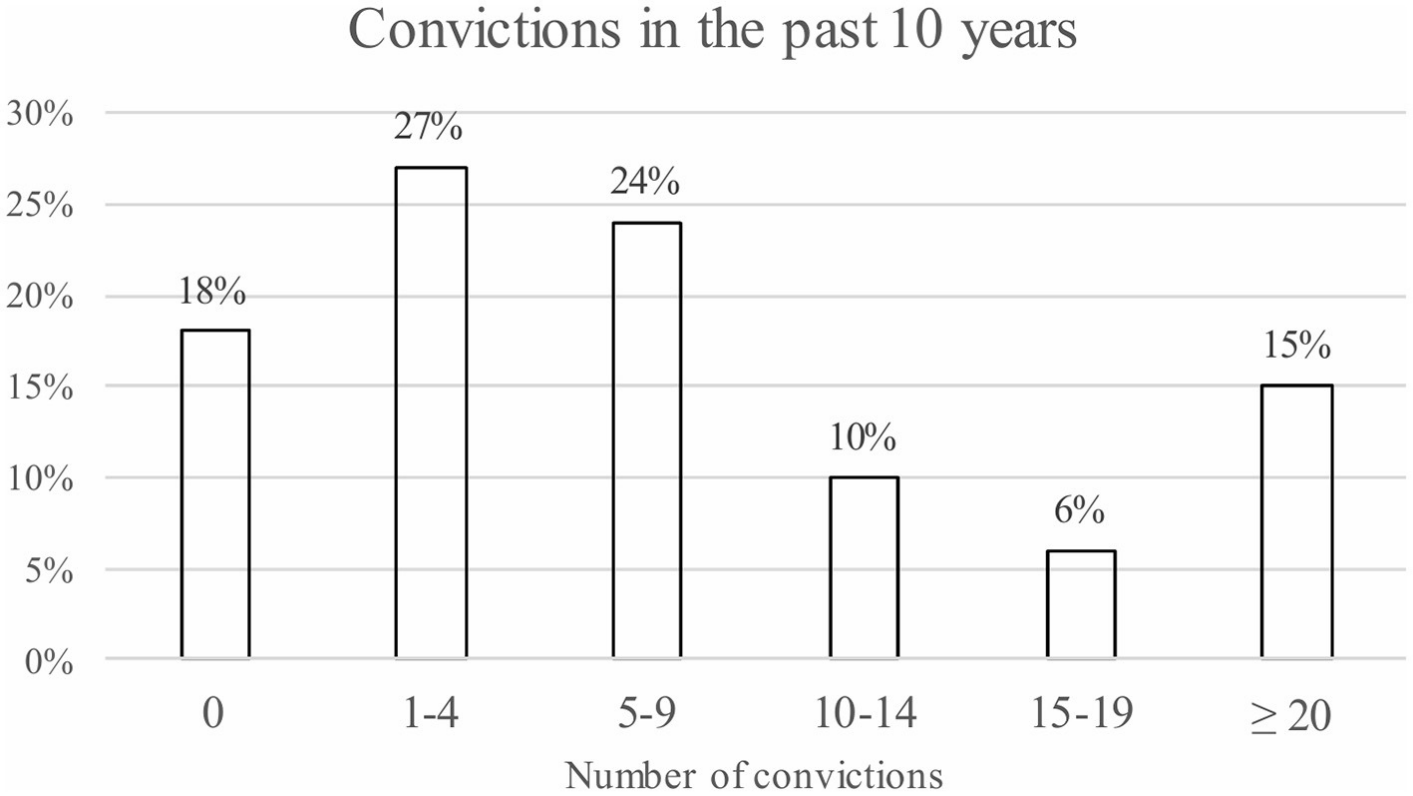
An overview of the number of convictions of the ForFACT patients in the past 10 years (*n* = 132).

Of the participating ForFACT patients, 5% had no criminal record, meaning they had no previous convictions, dismissed cases, mistrials, or acquittals in their past. In addition, 13% of the patients had no convictions, dismissed cases, mistrials, or acquittals in the past 10 years. However, these patients did have a criminal record and could have had one or more convictions before this time period (though these data were not included in the current study). On average, patients with a criminal record had 12 convictions (dismissed cases, mistrials, and acquittals not included) in the past 10 years (*M* = 11.9, *SD* = 13.7). There was a weak significant association between the clinical estimation of the risk to reoffend on the FORE and the number of convictions in the past 10 years [*r*_*s*__(111)_ = 0.28, *p* = 0.001]. On average, the subgroup of patients with a high to very high risk to reoffend had more convictions in the past 10 years (*M* = 13.6, *SD* = 14.7) than the subgroup of patients with a very low to moderate risk to reoffend (*M* = 7.0, *SD* = 11.2) [*U*_(111)_ = 990.5, *p* = 0.002]. Figure 3 displays how many actual convictions patients had during this period. With respect to the type of criminal offense: 55% of the patients committed a violent offense in the past 10 years, followed by a financial offense (47%), and vandalism or public order offenses (42%).

### Care

Most patients were referred to ForFACT through their general practitioner (47%), followed by a probation officer (35%), a general mental health care facility (12%), and a forensic mental health care facility (6%). More than one-third of the patients (34%) already had a previous referral to Kairos (ForFACT or outpatient clinic). The participating patients could have received ForFACT with (40%) or without (60%) a decision of the court.

Most ForFACT patients received treatment in their own living environment (63%). The other patients saw their ForFACT practitioner at the outpatient clinic (19%) or received a combination of treatment at home and at the outpatient clinic (19%). On average, patients were receiving ForFACT for 17 months, at the moment the electronic patient records were studied (*M* = 17.4, *SD* = 12.8). Some patients received treatment for 3 years or longer (8%).

During the intake procedure, patients are asked about their care needs at the beginning of treatment. Some patients were not able to formulate a personal care need. The practitioners define treatment goals for specific patients based on the information gathered during the intake procedure. The care needs defined by patients and treatment goals defined by practitioners were categorized and are shown in Table 1. Most patients reported to need help regarding psychological care (53%). In addition, more than half of the patients had a treatment goal related to psychological care (53%). In almost one-third of the cases (30%) there was a correspondence, meaning that the needs formulated by the patient matched the treatment goals specified by the professional.

**Table 1:** The self-expressed care needs of patients and treatment goals of practitioners at the beginning of treatment, divided into categories.

**Category**	**Care need defined**	**Treatment goal defined**	**Corresponding care**
	**by the patient**	**by the practitioner**	**needs—treatment goals**
Psychological care	53%	53%	30%
Impulse-control or aggression regulation	44%	52%	34%
Practical matters	34%	43%	19%
Social contacts	14%	30%	6%
Crime prevention	9%	15%	4%
Commitment and motivation	–	22%	–
Medication	6%	22%	5%
Daytime activities	5%	19%	2%
Substance use or addiction	5%	20%	3%
Training skills and abilities	–	20%	–
Other	8%	14%	3%
None	3%	–	–

*Corresponding means that both the patient and the practitioners respectively formulated a care need and a treatment goal within the same category*.

When looking at the type of care patients received in the past 10 years, before they were referred to ForFACT, more than half of the participating patients (52%) had a type of housing assistance in the past. In addition, 79% received outpatient treatment and 51% of the patients received inpatient care. On average, patients had 4 types of treatments in the past 10 years (before they were referred to ForFACT) (*M* = 4.2, *SD* = 2.5). A few patients (3%) had no previous treatment. There was a weak significant association between the number of diagnoses of a patient and the number of previous treatments (before ForFACT) [*r*_*s*__(114)_ = 0.20, *p* = 0.018]. Taken all together, patients indicated that they had an equal amount of positive, negative as well as neutral (neither positive nor negative) experiences with their received care in the past.

## Discussion

The main goal of this exploratory study was to gain insight into the characteristics of the complex ForFACT patient population, their (criminogenic) needs, and responsivity. Data were collected from 132 patients who received treatment from a Dutch ForFACT team. The results will largely be discussed by means of the three principles of the RNR model: risk, need, and responsivity (Andrews et al., 1990).

Estimating and monitoring the recidivism risk of patients is, according to the risk principle of the RNR model, of great importance. The results shows that the majority of ForFACT patients had a criminal record and a moderate to very high risk to reoffend during the intake procedure. However, one-third of the patients had a very low to low recidivism risk at the beginning of treatment. Given the type of care ForFACT offers, one would expect the recidivism risk, as well as the average scores on the dynamic risk factors, of these patients to be higher. In addition, several patients had no criminal record or no convictions in the 10 years before ForFACT. It should be noted that patients with no convictions within the past 10 years could have committed one or more (serious) offenses before this time period, followed by a long period of incarceration (during which no new crimes were committed). In addition, it is likely that several patients have not officially been convicted but have committed offenses without them being reported to the police (for instance, domestic violence). Lastly, some patients without a criminal record might show a (high) risk of potential criminal behavior.

These results raise the question whether patients with a low recidivism risk and/or no criminal record should receive ForFACT, or whether they should be treated by a regular flexible ACT team. Within the Dutch treatment context, patients can, under specific conditions, be referred to ForFACT without an order of the court (e.g., our results show that almost half of the patients are referred to ForFACT by their general practitioner, in the absence of a court order). ForFACT may experience pressure from general mental health facilities to accept non-forensic patients who have no previous or recent convictions, but who display aggressive/disruptive behavior (e.g., incidents toward staff, failing to keep agreements, or nuisance). Moreover, ForFACT experiences problems when referring patients with a reduced recidivism risk to less intensive/outreaching types of care, because these services are not keen on accepting patients with forensic features and aggression/impulsivity problems. This could explain why patients with a low risk to reoffend and/or no criminal record do receive ForFACT. Another possible explanation could be that these patients are admitted to ForFACT, not because of a current high risk of reoffending, but because of complex (externalizing) psychiatric needs, combined with severe responsivity issues. Apparently, there is a need for a specialized service like ForFACT, with forensic expertise, to treat these types of patients.

According to the RNR model, forensic psychiatric care should focus on the needs of patients in order to be effective. The results related to diagnostics indicate that the needs of ForFACT patients vary and are highly complex. ForFACT patients suffer from multiple psychiatric disorders, of which the most common primary diagnoses are impulse-control disorders or personality disorders. Impulsivity seems to be an important distinctive characteristic of this patient population. Patients with impulse-control disorders and personality disorders may not be easily referred to flexible ACT teams, but are referred to ForFACT instead, because of their aggression/impulsivity, imminent criminal behavior and nuisance. When patients receive medication for their psychological problems, medication adherence seems to be a problem. In addition, several patients show a high risk of suicide and more than half of the ForFACT patients experience medical/somatic problems. Furthermore, the great majority of ForFACT patients experience addiction problems during ForFACT and/or have experienced problems with addiction in the past. In order for ForFACT to be effective, according to the RNR model, these various complex care needs should be assessed and addressed [especially if they influence/are related to (re)offending].

An issue underlining the complexity of the needs of ForFACT patients is that, in most cases, the self-expressed health care needs of patients during the intake procedure, and the treatment goals formulated by practitioners do not correspond. Some patients find it difficult to formulate and communicate their care needs, for instance, because of a limited understanding of their (psychiatric) problems, a lack of self-reflection, and/or diminished cognitive functioning. Improving the correspondence between the practitioner's treatment goals and the patient's needs expressed at the beginning of treatment is of importance for treatment motivation, treatment effectiveness and the patient's recovery. Determining the care needs of ForFACT patients seems to be far from easy. This might also be reflected in their complex history of care. The great majority of patients received multiple types of (unsuccessful) treatment in the past, before they are referred to ForFACT.

Besides as an expression of the heterogeneous and not easy to influence needs of ForFACT patients, this history of care is also related to another RNR principle: responsivity. There are numerous responsivity issues within this specific population that might influence treatment effectiveness. In this study, we zoomed in on a few of these issues. For instance, approximately one out of five patients show limited motivation and commitment for treatment. Especially such a group of patients may benefit from an outreaching type of care, which is a distinct feature of ForFACT. Another issue concerning responsivity is highlighted by the results of the cognitive screening. These results indicate that the majority of patients were qualified for further diagnostic research because of a (strong) suspicion of MCI or an intellectual disability. Insight into the intellectual and cognitive level of functioning of patients is of great importance for tailoring care and achieving treatment goals. Cognitive problems and intellectual disabilities may be associated and possibly interact with psychopathology, impulsivity and limited inhibition. ForFACT practitioners need to adjust the way they approach a patient to the patient's level of cognitive functioning. In addition, the level of cognitive functioning of patients may determine which type of collaboration is needed with other mental health services specialized in care for patients with intellectual disabilities, to provide the best type of treatment and continuity of care for these patients.

Even though this research study has been prepared and conducted thoroughly, some limitations should be mentioned. First, 41% of the approached ForFACT patients declined to participate. Although such percentages are far from unusual when conducting research within a forensic psychiatric population, this research project misses data on a (potentially) important group of ForFACT patients. Unfortunately, there is no information available about the characteristics of these patients because of privacy issues. Secondly, the information that was gathered from the interviews is subjective to the patient's ability to remember events from the past and should be interpreted carefully. Some patients indicated they could not remember exact dates, years, locations or types of treatment. Additionally, the sum scores of patients on the screening instruments is a contemporary snapshot of cognitive abilities and might be under the influence of external factors [e.g., psychological problems, emotional instability, medication, substance use, lack of daytime activities, and being unemployed (for a longer period of time)]. Furthermore, the administered instruments are designed for screening, not for determining a diagnosis, and there are no specific norms or cut-off scores available for the ForFACT patient population. With regard to the FORE, the estimation of the recidivism risk might not be completely accurate. During the course of treatment, practitioners will get to know a patient and his/her circumstances better, which will probably also improve the accuracy of a FORE score. In addition, this study examined only two ForFACT teams. It may be possible that ForFACT teams across the Netherlands vary depending on the region (e.g., rural vs. urban), specific local situations/policies leading to potential differences in whom they treat and how they treat them. Finally, in order to cope with the amount of collected data in this study, categories were formed for the variables. There is always a small margin of error when categorizing variables. However, the inter-rater reliability for the categorized variables was studied and found to be substantial enough to proceed.

Besides the mentioned limitations, this study is one of the first studies that extensively looked into the characteristics of the ForFACT patient population. Based on the results it can be concluded that many patients adhere to the ForFACT criteria: they show a (high) risk of (re)offending, have clear forensic care needs and display critical responsivity issues. However, it seems there is also a group of ForFACT patients who do not show a high risk of reoffending and have no criminal record. Initially, these patients do not appear to have specific criminogenic needs for forensic treatment. However, it seems as if they are in need of care within the scope of ForFACT because of their complex psychiatric care needs, aggression, disruptive behavior and responsivity issues (e.g., lack of commitment and motivation, diminished cognitive ability), which are often too complex for a regular flexible ACT team. This is in line with Kusters et al. (2018) who found that, for ForFACT patients, other factors (clinical factors) seem to have a more central role at predicting (re)offending than criminogenic factors within the START. The majority of the ForFACT patients' treatment goals are defined within the categories of psychological care, impulse-control or aggression regulation, and practical matters. In addition, an international literature review indicated that FACT patients are not necessarily different from ACT patients, but their complex needs are (Cuddeback et al., 2020).

Besides providing care for patients with criminal charges, ForFACT seems to operate on the interface between forensic mental health care and general mental health care by also providing care for a group of patients with complex needs and responsivity issues (according to the RNR model), but without a high risk of (re)offending. This group is at risk of receiving no treatment at all or being referred to different settings, as professionals struggle to find the correct approach for the complex needs and responsivity issues of these patients. For this group of patients, ForFACT acts in a preventive manner by focusing on treating the needs of these patients in order to prevent actual offending. Furthermore, it seems as if regular flexible ACT teams are not able to fully respond to the responsivity problems and these patients are therefore often referred to ForFACT (e.g., by their general practitioner as well as other mental health services). The decision within the Netherlands to let ForFACT also treat patients with a risk of criminal behavior, but no current conviction, may raise questions about whether this is the most effective method. One could argue that, for the time being, these patients do receive treatment and are therefore not stuck between mental health care and forensic psychiatric care. Still, it is very important to continually check inclusion and exclusion criteria when admitting patients to ForFACT, and to check whether ForFACT or regular flexible ACT is the best-indicated type of care. A distinct feature of ForFACT is its ability to provide continuity of care by (temporarily) intensifying or de-intensifying treatment. It is important to prevent patients from receiving unnecessary prolonged treatment by continually monitoring the possibilities of patients to be referred to a less intensive/outreaching type of care. In line with Cuddeback et al. (2020), ForFACT needs to include clear discharge and transition strategies, especially for patients who experience problems on the border of general and forensic care.

Due to responsivity issues, it can take a long time before a solid therapeutic relationship is build. The outcomes of this study therefore emphasize the importance of ForFACT teams to include practitioners with sufficient experience and skills within the forensic psychiatric field. This recommendation is in line with Cuddeback et al. (2020). In addition, the collaboration of ForFACT with other mental health institutions is of great importance to correspond to the complex and various needs of ForFACT patients, and to provide them with tailored care. For example, considering that the vast majority of patients experience problems related to substance abuse, a ForFACT team should have enough knowledge and expertise on addiction, and collaborate with specialized addiction treatment centers. Since many patients may be referred to ForFACT by (high) security settings, the collaboration with these settings and supervising forensic or criminal justice institutions is also very important. Lastly, the results of this study highlight the importance of cognitive screening for forensic outpatients, as well as the importance of sufficient expertise and the possibilities of ForFACT practitioners to adjust care depending on the patient's level of cognitive functioning.

Thanks to this study, more knowledge is available about the ForFACT patient population. However, the results provided by this study raise additional research questions, for instance, regarding the upscaling and downscaling of treatment. Future research could focus on multiple single case studies to test the effects of the ability of ForFACT to (temporarily) (de)intensify treatment. It would also be interesting to compare the patient characteristics of the ForFACT population and the regular flexible ACT population, and to zoom in on the group of patients who experience problems within the interface of forensic care and general mental health care. Furthermore, more knowledge is needed about the type and severity of the disruptive behavior and possible associated risks, needs and responsivity issues, which causes patients to be referred to ForFACT. Future research could look into discharge and referral processes between different types of (forensic) mental health care facilities in order to improve these processes, and to make sure patients receive the most appropriate type of care.

## Data Availability Statement

The raw data supporting the conclusions of this article will be made available by the authors, without undue reservation.

## Ethics Statement

The studies involving human participants were reviewed and approved by The Internal Scientific Committee of the Pompefoundation. The patients/participants provided their written informed consent to participate in this study.

## Author Contributions

MS and FS performed the data collection and organized the dataset, by supervision of MV and BB. MS performed the statistical analyses en wrote the first draft of the manuscript. All authors contributed to the conception and design of the study, manuscript revision, read, and approved the submitted version.

## Funding

This study was funded by Koers en Kansen voor de sanctie-uitvoering, a program set out by the Dutch ministry of Justice and Security, and the Pompefoundation.

## Conflict of Interest

The authors declare that the research was conducted in the absence of any commercial or financial relationships that could be construed as a potential conflict of interest.

## Publisher's Note

All claims expressed in this article are solely those of the authors and do not necessarily represent those of their affiliated organizations, or those of the publisher, the editors and the reviewers. Any product that may be evaluated in this article, or claim that may be made by its manufacturer, is not guaranteed or endorsed by the publisher.
